# Integrating multi-modal remote sensing, deep learning, and attention mechanisms for yield prediction in plant breeding experiments

**DOI:** 10.3389/fpls.2024.1408047

**Published:** 2024-07-25

**Authors:** Claudia Aviles Toledo, Melba M. Crawford, Mitchell R. Tuinstra

**Affiliations:** ^1^ Lyles School of Civil Engineering, Purdue University, West Lafayette, IN, United States; ^2^ Department of Agronomy, Purdue University, West Lafayette, IN, United States

**Keywords:** hyperspectral, LiDAR, stacked LSTM, attention mechanisms, multi-modal networks, yield prediction, precision agriculture

## Abstract

In both plant breeding and crop management, interpretability plays a crucial role in instilling trust in AI-driven approaches and enabling the provision of actionable insights. The primary objective of this research is to explore and evaluate the potential contributions of deep learning network architectures that employ stacked LSTM for end-of-season maize grain yield prediction. A secondary aim is to expand the capabilities of these networks by adapting them to better accommodate and leverage the multi-modality properties of remote sensing data. In this study, a multi-modal deep learning architecture that assimilates inputs from heterogeneous data streams, including high-resolution hyperspectral imagery, LiDAR point clouds, and environmental data, is proposed to forecast maize crop yields. The architecture includes attention mechanisms that assign varying levels of importance to different modalities and temporal features that, reflect the dynamics of plant growth and environmental interactions. The interpretability of the attention weights is investigated in multi-modal networks that seek to both improve predictions and attribute crop yield outcomes to genetic and environmental variables. This approach also contributes to increased interpretability of the model's predictions. The temporal attention weight distributions highlighted relevant factors and critical growth stages that contribute to the predictions. The results of this study affirm that the attention weights are consistent with recognized biological growth stages, thereby substantiating the network's capability to learn biologically interpretable features. Accuracies of the model's predictions of yield ranged from 0.82-0.93 *R^2^_ref_* in this genetics-focused study, further highlighting the potential of attention-based models. Further, this research facilitates understanding of how multi-modality remote sensing aligns with the physiological stages of maize. The proposed architecture shows promise in improving predictions and offering interpretable insights into the factors affecting maize crop yields, while demonstrating the impact of data collection by different modalities through the growing season. By identifying relevant factors and critical growth stages, the model's attention weights provide valuable information that can be used in both plant breeding and crop management. The consistency of attention weights with biological growth stages reinforces the potential of deep learning networks in agricultural applications, particularly in leveraging remote sensing data for yield prediction. To the best of our knowledge, this is the first study that investigates the use of hyperspectral and LiDAR UAV time series data for explaining/interpreting plant growth stages within deep learning networks and forecasting plot-level maize grain yield using late fusion modalities with attention mechanisms.

## 1 Introduction

Plant breeding experiments play a critical role in the development of future generations of crops that can effectively respond to the increasing global food demand and the impact of climate change (Razzaq et al., 2021). Using advanced technologies, such as remote sensing (RS) and machine learning, plant breeders and researchers seek to make more informed decisions regarding their crops (Akhter and Sofi, 2022). By including genetic information and environmental inputs such as soil properties and weather patterns, predictive models can now forecast future yields and rank new hybrids with increased precision. Use of advanced predictive models has significantly altered the approach of researchers toward the development of new crop varieties in maize breeding experiments (Xu et al., 2022). These predictive tools significantly accelerate the breeding process, allowing researchers to focus their efforts on the most promising candidates, thus increasing the rate of development of high-yielding and resilient varieties. Improved prediction of end-of-season traits in the field can also allow preliminary selection of the most promising individuals based on RS plant phenotyping at early developmental stages (Wang et al., 2023).

Maize is an annual grass species, completing its life cycle within one growing season (Eckhoff et al., 2003). Using RS, it is possible to model development of maize through the growing season by acquiring data during different stages of physiological development, thereby creating a time series. High-throughput phenotyping provides capability for monitoring and assessing crop growth and plant attributes. The accessibility of these technologies has increased due to recent advances in sensors and platforms. This provides breeders with the opportunity to explore larger datasets to examine the relationships between genetics, environment, and management practices. The Genomes 2 Fields (G2F) project is a multi-university research initiative aimed at enhancing the productivity and sustainability of crops by integrating genomics and field-based breeding efforts (AlKhalifah et al., 2018). In these experiments, the use of doubled haploids is intended to serve as an effective means of accelerating the breeding process, validating genomic discoveries, enhancing specific traits, and conserving genetic diversity, thus contributing to the development of resilient and high-yielding crop varieties (AlKhalifah et al., 2018). However, the small number of replicates of the same doubled haploid hybrids leads to a restricted portrayal of the phenotypic traits of the variety when training models. This issue was resolved in this study by utilizing publicly available genetic data, clustering similar genetic groups of varieties, and implementing stratified sampling during the training process.

Long-Short-Term Memory (LSTM) networks, a form of recurrent neural network (RNN), have recently demonstrated efficacy in handling time series data, including in agronomical scenarios. Previous studies using this network architecture have achieved high accuracy in crop yield prediction (Masjedi et al., 2020; Khaki et al., 2021; Wang et al., 2023). Attention mechanisms have been investigated to enhance model accuracy, and have also demonstrated their effectiveness in improving model interpretability (Gangopadhyay et al., 2020; Danilevicz et al., 2021; Tian et al., 2021; Toledo and Crawford, 2023). Modeling based on multi-modal RS data has also been studied, primarily exploring early fusion (Masjedi et al., 2020; Wang and Crawford, 2021). Early fusion involves combining different modalities at the beginning of the processing pipeline, i.e., as integrated, normalized inputs to a model (Wang et al., 2020a). However, drawbacks of these multi-modal RS-based LSTM prediction models are: i) simultaneous representation of both internal and external interactions among the modalities; ii) reduced understanding and interpretability of the predicted results; iii) less capability to explore the connection between the physical growth stages and their relationship with the time series being modeled.

In this study, three LSTM-based architectures (vanilla stacked LSTM, stacked LSTM with a temporal attention mechanism and multi-modal attention networks) are investigated in plot-level end-of-season maize yield prediction experiments using multi-modal RS data and weather data. Time-step importance is first evaluated using the time domain attention weights for each modality to investigate the impact of each sensor-based input during the growing season. Based on sensitivity analysis of the time-steps provided by the temporal attention weights, multiple scenarios are explored, where different growth stages within each modality are considered. The scenarios are investigated in all three proposed architectures. Data from a two-year GxE experiment of doubled haploids using the same tester parent was used to evaluate the proposed objectives. The paper is organized as follows: Related Work provides a review of deep learning prediction models that have RS data inputs, emphasizing attention mechanisms and multi-modal networks; Materials and Methods includes a description of the study sites, datasets, and the methodology; Results are presented and discussed in Experimental Results; Conclusions and recommendations for future work are summarized in Conclusions and Discussion.

## 2 Related work

### 2.1 Yield prediction models

Regression models based on inputs, including RS data, weather, soils, genetics, and management practices, have been widely investigated in agriculture for yield prediction. Early studies based on multiple regression were followed by classical machine learning approaches, including support vector regression (SVR), partial least squares regression (PLSR), and random forests (RF) to predict grain yield and biomass, respectively (Sujatha and Isakki, 2016; Masjedi et al., 2020). (Aghighi et al., 2018) incorporated RS time series data into classical machine learning models, such as boosted regression trees (BRT), RF, and SVR. Traditional machine learning-based models are difficult to generalize to scenarios outside the domain of the training data. Furthermore, these models lack the ability to effectively leverage inputs from time series data across multiple modalities or differentiate between categorical variables and time series data, such as environmental conditions (soils), management practices, or genetic inputs. Furthermore, they do not incorporate time series data into a step-wise framework, which is crucial for simulating the growing season and comprehending prediction outcomes. More recently, yield prediction models have been developed using deep learning architectures, particularly at large-spatial scales (Maimaitijiang et al., 2020; Khaki et al., 2021; Shook et al., 2021). These architectures have recently been investigated to predict yield using RS data as inputs (You et al., 2017; Wang et al., 2020c). (Jiang et al., 2020) developed an LSTM framework using MODIS remote sensing products to predict county level yields. At the research plot scale, (Masjedi et al., 2019; Wan et al., 2020; Wang and Crawford, 2021) used RS data acquired by UAV platforms to predict yields in sorghum and maize. (Wang and Crawford, 2021) extended this work to investigate transfer learning of models to other locations and time periods. Despite these advances, further improvement is needed in predictive models to leverage multiple modality RS and, most importantly, achieve interpretability in the predicted outcomes.

#### 2.1.1 LSTM-based yield prediction models

The application of recurrent neural networks (RNN) has led to the emergence of robust learning models, characterized by interpreting complex or abstract features to derive meaningful patterns from the inputs (Lipton et al., 2015). As with many neural network architectures, the relationship between features is established through multi-level hierarchical representations, which enables them to extract features and learn from the datasets (LeCun et al., 2015). Long-term short-memory (LSTM) based networks were developed to address the well-known vanishing gradient problem of RNNs (Gamboa, 2017). The core architecture of these networks includes memory cells, referred to as LSTM cells, whose purpose is to store the data and update it through forget, input and output gates (Kong et al., 2019). LSTM has become popular in long-term temporal time series predictions, such as crop yields. The majority of studies concentrate on yield predictions at county or regional levels. (Tian et al., 2021; Chen et al., 2023) used satellite time series data based on an LSTM model on time accumulated data for wheat yield predictions. (Sun et al., 2019) examined the performance of CNN, LSTM, and CNN-LSTM architectures for predicting soybean yield at the county level. Small-scale experiments with high resolution data have also demonstrated the advantages of LSTM models. (Masjedi et al., 2019) studied an LSTM-based RNN model using multi-temporal RS data to predict fresh sorghum biomass. (Shen et al., 2022) utilized both LSTM and LSTM-RF architectures with UAV thermal and multispectral imagery to forecast wheat yield at the plot level. Although all these studies produced results with R^2^ values ranging from 0.60-0.94, they lack interpretability regarding the growing season.

#### 2.1.2 Attention mechanisms

Attention mechanisms were first introduced to address the problem of information overload in computer vision (Itti et al., 1998). In image classification, attention mechanisms were incorporated in a neural network to extract information from an image by adaptively selecting a sequence of spatial regions and focusing on these regions at high resolution (Mnih et al., 2014). The attention models were introduced in machine-based text translation tasks by (Bahdanau et al., 2016) to distribute information of the source sentence across all sequences, rather than encoding all the information into a fixed-length vector through the encoder. Attention mechanisms have commonly been categorized as: spectral/channel, spatial, and temporal attention (Guo et al., 2022). The concept of spectral attention focuses on recalibration of channel weights and their interrelationships, thereby enhancing their representation (Hu et al., 2018). Given the high dimensionality and redundancy in adjacent spectral bands, spectral attention is commonly employed in hyperspectral image classification. The concept of temporal attention mechanisms originated in video processing, providing a dynamic method of determining “when and where” attention should be directed (Li et al., 2020). In time series sequences, the decoder can selectively retrieve the focused sequence at each time-step. Temporal attention mechanisms seek to localize important parts of the input features in the time dimension through attention weights from earlier time-steps. Attention weights represent a distribution over input features, providing a tool for interpretation (Serrano and Smith, 2019). Temporal attention enhances the inherit function of LSTM cells of capturing long time dependencies by identifying the time-steps relevant to the prediction and extracts the information from these time-steps (Shih et al., 2019). Some recent studies have investigated yield prediction attention networks in multiple crops using multispectral satellite data, focusing on the environmental component of GxE (Khaki and Wang, 2019; Gangopadhyay et al., 2020; Shook et al., 2021). Although integration of medium resolution multispectral data for county-level yield predictions has been studied (You et al., 2017; Wan et al., 2020), use of temporal attention mechanisms in conjunction with LSTM’s with high-resolution UAV inputs for small plot breeding trials has not been previously explored to the best of our knowledge.

#### 2.1.3 Multi-modal deep learning

Multi-modal deep learning involves training deep neural networks to extract and learn features from multiple types of data. The core concept of multi-modal feature learning is that including multiple data modalities enables more effective learning of one modality compared to in-depth feature extraction from a single modality. (Wang et al., 2020a) investigated voice and text-based fusion to improve the effect of emotion recognition (Liu et al., 2021). Recently, in time series predictions (Xian and Liang, 2022) used holidays, weather data, and quarterly market operation information reports in multi-modal networks to predict traffic conditions. Development of maize crops is strongly influenced by both genetic and environmental factors, motivating their inclusion in models when the data are available. Several studies have employed multiple types of RS data as input for DL models to predict crop yield (Danilevicz et al., 2021; Shook et al., 2021; Wang and Crawford, 2021; Toledo et al., 2022). Multi-modal deep learning models characterize and learn from different sources of input data. For example, LiDAR represents structural attributes of maize through the growing season, while hyperspectral data are related to chemistry related responses of the plant. The information represented by different modalities can potentially be leveraged in a combined model for a better representation of the task at hand (Kumar et al., 2022). (Maimaitijiang et al., 2020) integrated canopy structure, temperature, and texture, training each modality individually with multiple CNN layers and applied late fusion for the yield prediction. Furthermore, they also tested an input-level feature fusion, incorporating multiple CNN layers, but the approach underperformed in comparison to the late fusion.

### 2.2 Multi-modal remote sensing

In agriculture, multiple RS technologies, including RGB, multi/hyperspectral, thermal cameras, and LiDAR (Ali et al., 2022) on airborne and space-based platforms have been used to assess crop properties. (Zhang et al., 2020) integrated optical, thermal, and environmental data to predict county-level maize yield, and focused on demonstrating that combining multi-modal, multi-source data explained the variation in yield. Hyperspectral sensors provide high spectral and spatial resolution data when flown on UAV platforms (Li et al., 2022). Many bands of the continuous, contiguous spectral data are highly correlated, motivating feature extraction and feature selection. In vegetation related studies, spectral indices are commonly used to represent important chemistry-based absorption features (Jain et al., 2007). Derivative and integral characteristics of hyperspectral cubes also represent important spectral changes in reflectance that can characterize the crop canopies (Masjedi et al., 2018). Many indices are also highly correlated, resulting in redundancy that may either lead to overfitting or weaken the predictive capability of deep learning models (LeCun et al., 2015). LiDAR point clouds provide geometric characteristics of the plants such as plant height, canopy cover, and canopy volume. Because of the large number of candidate features from hyperspectral and LiDAR data, (Toledo et al., 2022) investigated DeepSHAP, which uses Shapley values to quantify the contribution of each feature in a prediction made by a deep learning model.

## 3 Materials and methods

### 3.1 Plant breeding field experiments

The experiments reported in this study were conducted in Indiana, USA. The experiments were planted in different fields in 2020 and 2021 at the Agronomy Center for Research and Education (ACRE) at Purdue University (40°28’37.18”N, 86°59’22.67”W), West Lafayette. Both were planted in a randomized incomplete block design with two replications. The core check hybrids had two complete replications, and the doubled haploid hybrids based on the PHK76 tester had an incomplete block design.

**Figure 1:**
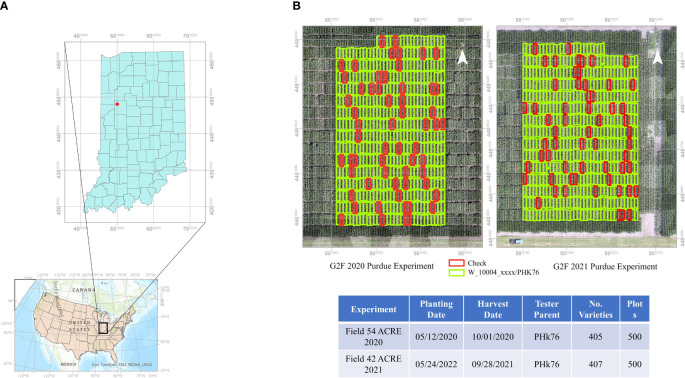
**(A)** Geographic location of maize experiments at Purdue University’s Agronomy Center for Research and Education. **(B)** Experimental plot layouts for GxE plant breeding experiments in 2020 and 2021. Check plots indicated in red.

The experiments were planted as two row plots with a length of 4.575 m by 1.5 m with ~76 cm row spacing. Standard nutrients, herbicides, and insecticides were applied according to normal agronomic management practices at the beginning of the season, and there was no artificial irrigation. Both fields were planted in an annual crop rotation with soybeans. They were planted on May 12, 2020, and May 24, 2021, at a population of 74,000 seeds ha^-1^, respectively. Anhydrous ammonia (NH3) was applied prior to planting in 2020 and liquid Urea Ammonium Nitrate solution (UAN) was applied in 2021. **Figure 1** shows the geographic location and layout of the field experiments. The grain yield was harvested from both rows on October 1, 2020, and September 28, 2021, using a Kincaid plot combine (Kincaid 8-XP, Haven, KS, USA) with grain yields adjusted to 15% moisture.

### 3.2 Genetics and ground reference data

**Figure 2:**
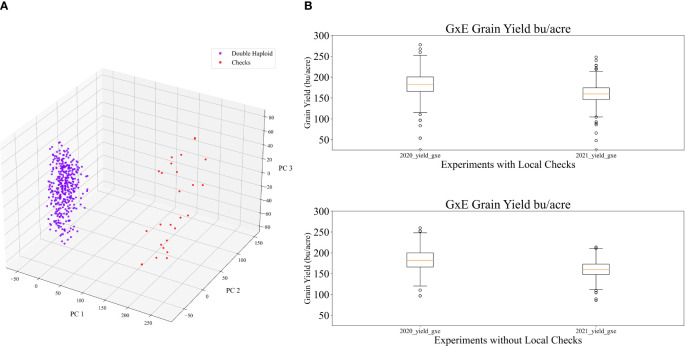
**(A)** Genetic variation based on PCA **(B)** Ground reference data with and without check data.

Because the G2F Initiative covers multiple environments and geographic locations, local core check hybrids are used as standards against which the performance of new breeding lines or varieties are compared. By evaluating the performance of new varieties relative to local checks, breeders can evaluate traits such as yield potential, disease resistance, and overall agronomic suitability (Ullah et al., 2017). Using local checks in multiple environments helps validate the data collected from experimental trials. The consistent performance of local checks in different environments instills confidence in the experimental setup, thus ensuring that the observed performance differences among the new breeding lines are significant and not influenced only by variation in environmental conditions. Because this study focuses on evaluating the performance of genetic variations of double haploids with the tester, local checks introduce an imbalance in terms of their genetic variation. The G2F provides a public genotypic data set in which inbred parents of the hybrids tested were genotyped using the Practical Haplotype Graph (PHG) (Genomes To Fields, 2023). Quality control on the initial raw genotypic dataset of the inbred lines used in this study was performed as described in (Tolley et al., 2023). The resulting genetic marker matrix had 142,568 genetic markers from 401 varieties (including local checks). The dimensionality of the genetic data was reduced via principal components (PCs) from the original genetic marker data. A scree plot, which displays the explained variance of the individual principal components, was employed in conjunction with the elbow method to determine the appropriate number of principal components to utilize (Cattell, 1966). In the preliminary stage, the contributions of twenty PCs were computed. As evidenced in **Supplementary Figure 1**, the elbow test indicates that 6 PCs represent the ideal number for representation of the genetic variation. Two genetic clusters were identified based on the first 3 PCs, as shown in **Figure 2**, one associated with the DH hybrids and the other the core check hybrids. The under-representation of local checks, which comprise less than ~5% of the hybrids, can contribute to decreased accuracy in yield prediction because of the limited dataset for learning. In order to evaluate the performance of genetic variations in double haploids using the tester, local checks were excluded from the training and testing datasets. The ground reference grain yield data serves as additional evidence to support this observation, as shown in **Figure 2**.

### 3.3 Remote sensing data

**Figure 3:**
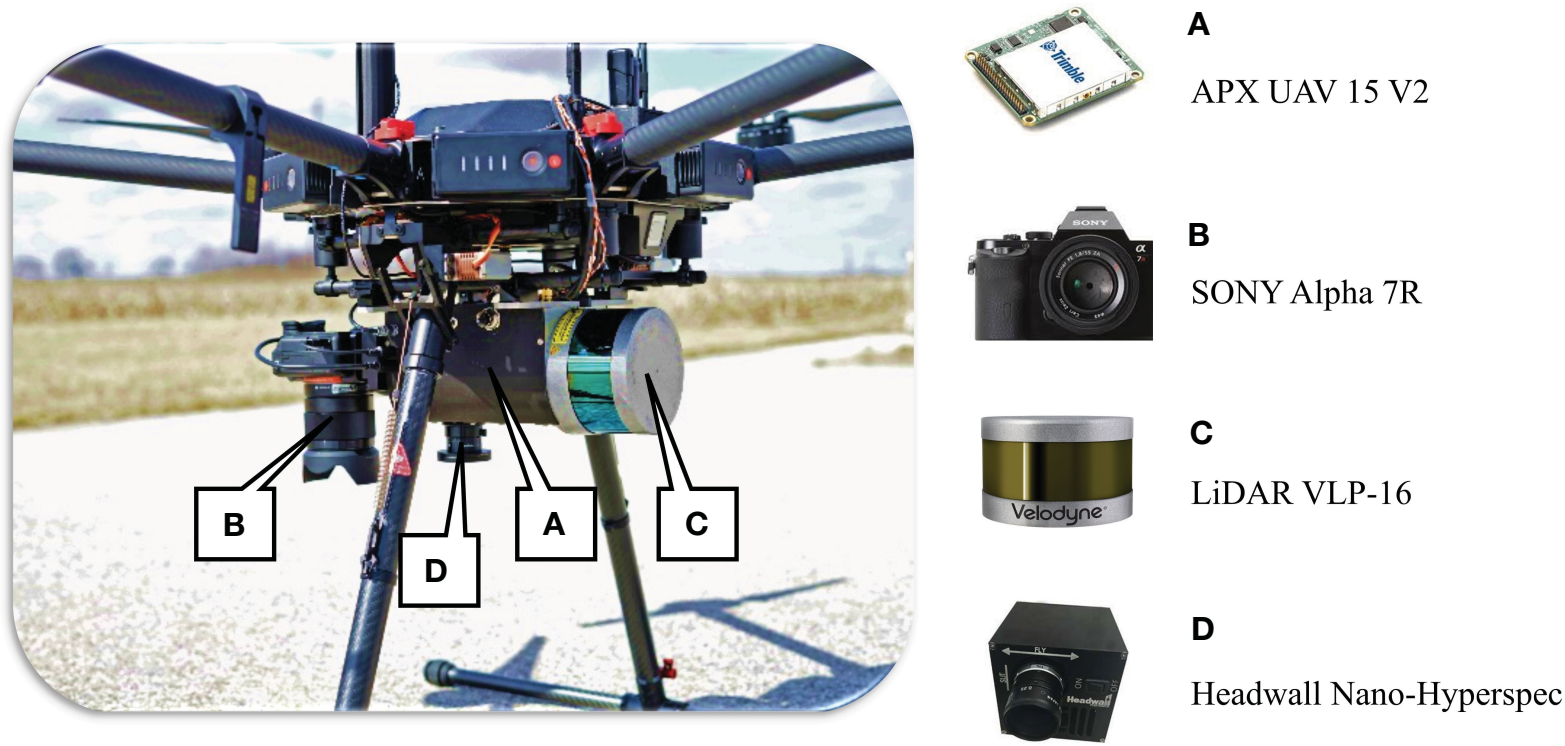
UAV platform with APX **(A)**, RGB **(B)**, LiDAR **(C)** and Hyperspectral **(D)** sensors.

The RS data were collected throughout the growing season in each study; each data collection was conducted in cloud-free conditions with calm winds. Data were acquired using a DJI multi-rotor Matrice 600 Pro (**Figure 3**), equipped with an Applanix APX-15v3 GNSS/IMU for accurate geo-referencing. An integrated sensor package comprised of three sensors was installed: (a) Nano-Hyperspec^®^ VNIR camera (Headwall Photonics Inc., Bolton, MA) with a spectral range of 400-1000 nm, 270 spectral bands at 2.2nm/band from 400 nm to 1000 nm with 640 spatial channels at 7.4 μm/pixel, flown at 44 m to achieve 4 cm spatial resolution in the final orthorectified cubes, (b) Velodyne VLP-16 Lite LiDAR sensor and (c) Sony Alpha 7RIII high resolution RGB camera. Rigorous system calibration was performed to estimate camera distortion and the relevant rotation angles and lever arms of the pushbroom sensor (Gharibi and Habib, 2018; LaForest et al., 2019). The specifications of the remote sensing data collection and its products are presented in **Table 1**.

**Table 1:** Data collection specifications.

Specifications	
Flying Height and Speed	44 m, 4.1 m/sec
Hyperspectral Ortho mosaic	4 cm (GSD) 4.4 nm (400 – 1000 nm) (spectral range)
RGB Orthophoto	0.5 cm
LiDAR Point Cloud and DSM	8 cm DSM

#### 3.3.1 Hyperspectral data

A dark current spectral response was collected at the beginning of each flight to allow conversion of raw DNs (Digital Numbers) to radiance using the absolute radiometric coefficients provided by the camera manufacturer. Three calibrated spectral targets (11%, 30% and 56%) were deployed for each UAV flight and used to convert radiance to reflectance values using the empirical line method. The hyperspectral imagery was orthorectified using the DSM derived from the LiDAR based georeferenced point clouds (Lin and Habib, 2021) and the methodology described in (Gharibi and Habib, 2018). Non-vegetation pixels were removed using the OSAVI value as a threshold (Li et al., 2016). The GxE experiment in 2020 is illustrated in **Supplementary Figure 2**, which includes the hyperspectral orthomosaic, OSAVI image, and non-vegetation pixel mask during flowering time.

#### 3.3.2 LiDAR and RGB data

The LiDAR point clouds were processed using the estimated mounting parameters aided by the GNSS/INS trajectory (Lin et al., 2019). For this study, the high resolution RGB orthophotos were used to extract the row/plots boundaries from each experiment, using the method described in (Yang et al., 2021). An example of the reconstructed LiDAR point cloud during flowering time is shown in **Supplementary Figure 3**.

#### 3.3.3 Dates for analysis

Data were collected throughout the growing season, with an effort to collect information every week. In the process of model development, careful consideration was given to the selection of dates to capture the essential temporal dynamics that impact crop yields. These dates and the related physiological stage of the plants are summarized in **Table 2**.

**Table 2:** Dates of remote sensing data acquisition in 2020 and 2021.

Data Type	Vegetative Stage	Field 54 2020		Field 42 2021	
		Experiment Dates	Growing Degree Days	Experiment Dates	Growing Degree Days
LiDAR & Hyperspectral	V8 V12 VT-R1 R1 R2 R4 R5	June 17^th^ July 2^nd^ July 17^th^ July 28^th^ August 6^th^ August 13^th^ September 5^th^	499 857 1222 1494 1660 1803 2288	June 17^th^ July 3^rd^ July 19^th^ July 27^th^ August 8^th^ August 16^th^ September 6^th^	703 1067 1444 1644 1895 2084 2589

#### 3.3.4 Feature extraction and feature selection

In each experiment, 40 cm was trimmed at each end of the rows to minimize human interactions, light differences, and treatments from neighboring plots. Both rows were used for the extraction of LiDAR and hyperspectral features, and subsequently averaged to derive the plot-based value. Initial candidate spectral features, including vegetation indices, integration, and derivative-based features, were investigated. The candidate LiDAR features were comprised of different percentiles of height, LiDAR canopy cover, volume and plot-based height statistical features (Masjedi et al., 2020). In accordance with Section 2.2, feature selection was conducted using the DeepSHAP methodology (Toledo et al., 2022). Nine hyperspectral features and seven LiDAR features were chosen as the remote sensing inputs for the time series analysis for both years. The detailed descriptions of these features are included in **Table 3**.

**Table 3:** Remote sensing input features for time series analysis in models.

Feature	Equation	Explanation
Hyperspectral		
Integration features of bands in the 670-780 nm bands	*Intg*(*λ_a_,λ_b_*) = $\int_{{\text{λ}}_{a}}^{{\text{λ}}_{\text{b}}}S(\lambda )d\lambda$ = area under the spectral curve for a given range [*λ_a_*, *λ_b_*]; where *S*(*λ*) is the reflectance *λ* nm.	Related to the increase in the NIR signature in the early season followed by a reduction after the maximum value, typically at flowering
Integration features of bands in the 910-1000 nm bands		
Integration of the first derivative of the NIR		
VOG3 (Vogelmann et al., 1993)	$\frac{\rho_{734}-\rho_{747}}{\rho_{715}+\rho_{720}}$	Chlorophyll related indices
NDRE (Barnes et al., 2000)	$\frac{\rho_{790}-\rho_{720}}{\rho_{790}+\rho_{720}}$	
MCARI2 (Daughtry, 2000)	$\left[(\rho_{750}-\rho_{705})-0.2(\rho_{700}-\rho_{550})]*(\frac{\rho_{750}}{\rho_{705}}\right)$	
DATT3 (Datt, 1999)	$\frac{\rho_{754}}{\rho_{704}}$	Chlorophyll related with high sensitivity to nitrogen
PSRI (Merzlyak et al., 1999)	$\frac{\rho_{678}-\rho_{500}}{\rho_{750}}$	Plant senescence index
RDVI (Roujean and Breon, 1995)	$\frac{\rho_{800}-\rho_{670}}{\sqrt{\rho_{800}+\rho_{670}}}$	Similar to NDVI, but less sensitive to the effects of soil and sun viewing geometry
LiDAR		
75^th^ height Percentile	Height of the non-ground points at *i^th^* percentile	Represents vertical distribution of the LiDAR points in each plot
90^th^ height Percentile		
Height Quadratic Mean	Square root of the mean of the squared heights $Q=\;\sqrt{\frac{x_{1}^{2}+x_{2}^{2}+\ldots +x_{n}^{2}}{n}}$	Represents the average LiDAR height squared at the plot level
Volume	Area of 8 cm x 8 cm resolution	Aggregated volume of voxel
Canopy Cover at 20^th^ percentile height	Fraction of points above specified percentile	Proportion of canopy above a specified height percentile
Canopy Cover at 50^th^ percentile height		
Canopy Cover at 75^th^ percentile height		

### 3.4 Weather variables

**Figure 4:**
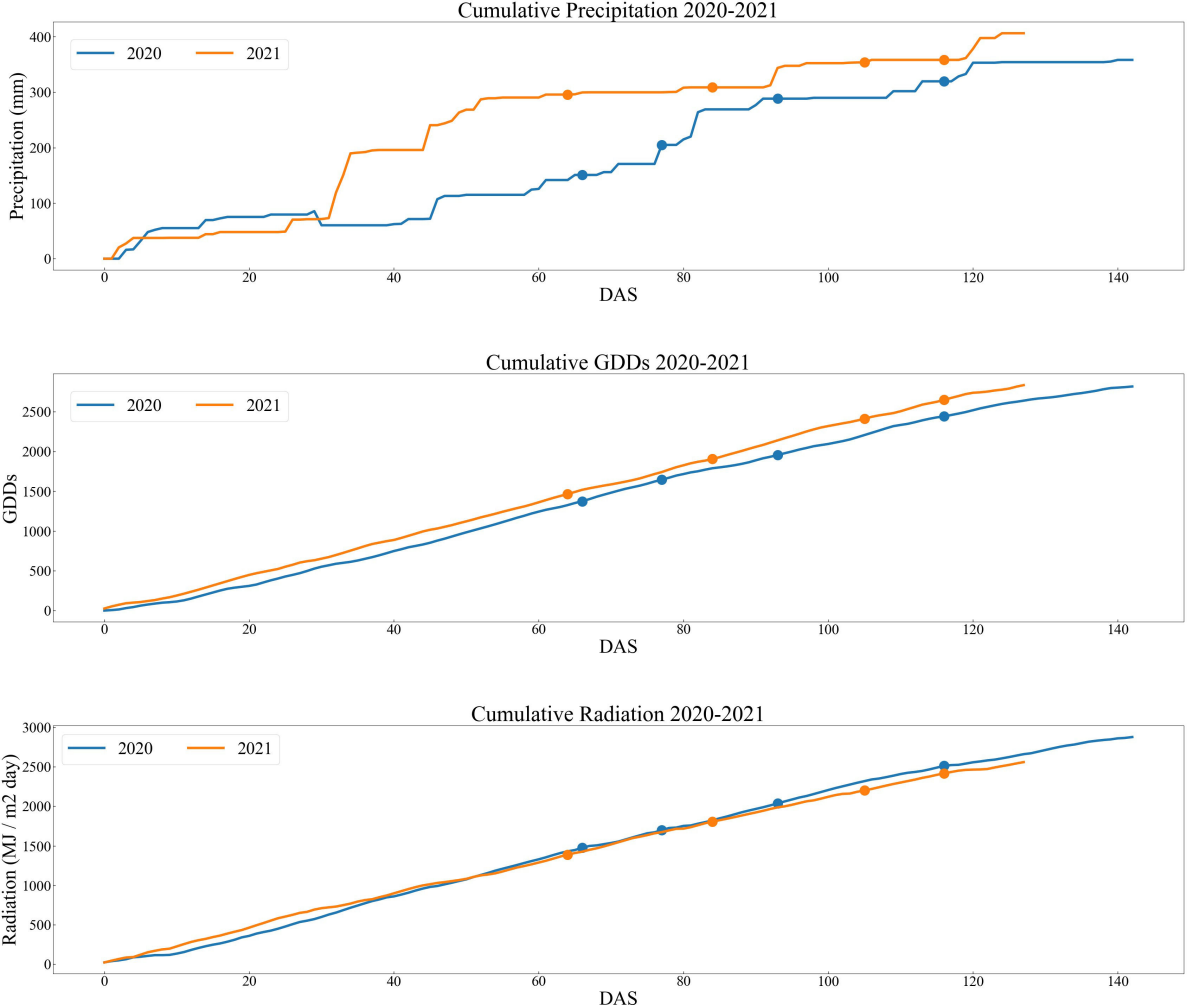
Accumulated values of weather variables through the growing season.

Three weather related variables were included in the analysis: Cumulative Radiation, Precipitation and Growing Degree Days (GDD) from the beginning of each of the growing seasons (2020-2021), shown in **Figure 4**. The precipitation and the growing degree days were obtained from the Indiana State Climate Mesonet weather station located at ACRE. All the data are publicly available at (Indiana State Climate Office, 2022).

### 3.5 Deep learning models of maize yield

**Figure 5:**
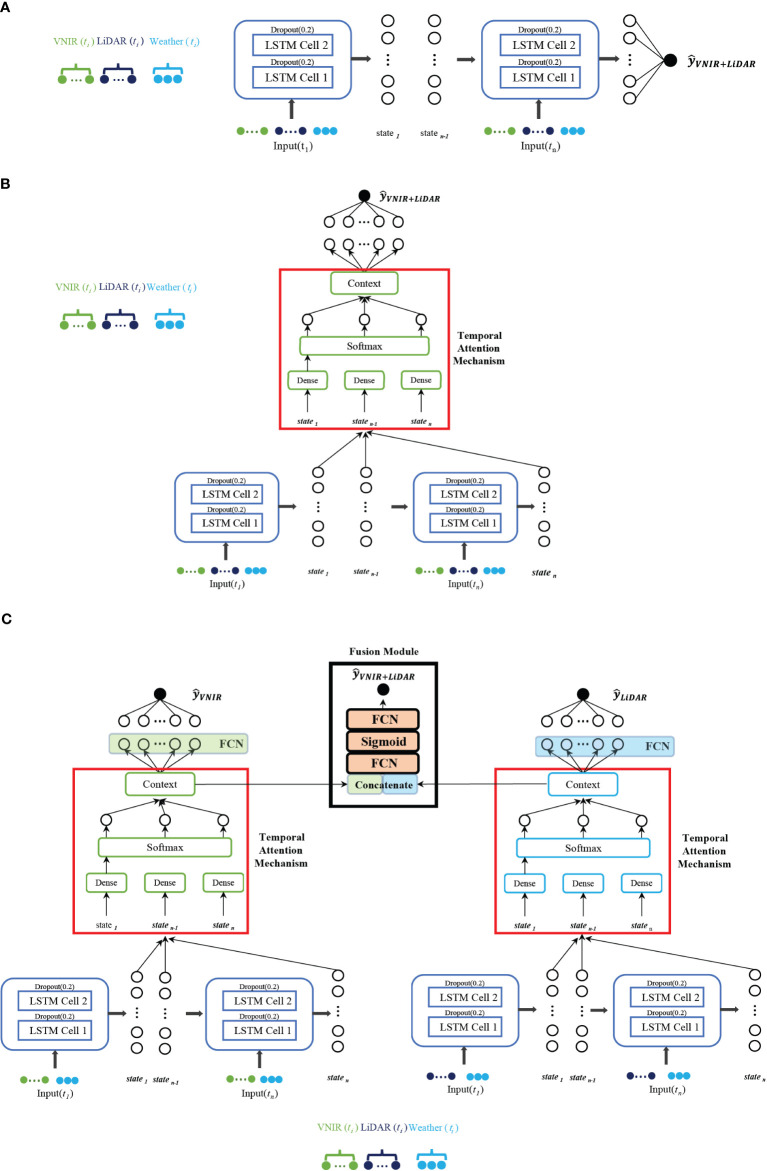
Stacked LSTM-based networks explored for prediction of maize yield ( ${\hat{y}}_{VNIR+LiDAR})$ . **(A)** Vanilla stacked LSTM with early fusion of both concatenated features from VNIR, LiDAR and weather data. **(B)** LSTM with attention mechanism, with early fusion of the modalities. **(C)** Multi-modal network with separate networks for each RS modality concatenated with weather, adding a late fusion module.

As noted previously, three RNN network-based architectures were implemented: a) vanilla stacked LSTM, b) stacked LSTM with an attention mechanism, and c) multi-modal network for the different RS modalities. For this study, the temporal attention mechanism was based on the Bahdanau attention mechanism (Bahdanau et al., 2016). **Figure 5** displays the stacked LSTM models described in the following sub-sections. The hyperparameters, including the use of the Adam optimizer (Kingma and Ba, 2017) for weight updating, were determined experimentally. The learning rate during training was set at 0.001. The Mean Squared Error (MSE) served as the loss metric for terminating model training. The model was developed using 5-fold cross validation with 80% training/20% testing and a 90/10 training/validation split of the training data for model development based on the 500 plots in each fold. All the networks were implemented in TensorFlow on an NVIDIA Quadro P400 GPU with 68 GB RAM.

#### 3.5.1 Vanilla stacked LSTM

The stacked network utilizes early fusion multi-modality, where the raw data from both modalities, along with weather data, are concatenated and input into the LSTM-based recursive neural network. The number of LSTM-cells was determined experimentally. A dropout layer of 0.2 was added after each LSTM cell to prevent over-fitting.

#### 3.5.2 Stacked LSTM with attention mechanism

The traditional LSTM network was coupled with an attention mechanism with a mid-season gate, which enhances performance and serves as a source of explainability. The attention mechanism computes a context vector that represents the relationship between the output of the hidden states of each time-step and each feature of the input vector (Niu et al., 2021), as depicted in **Figure 5B**. This comparison is typically accomplished using a weighted sum of a similarity score between the decoder state and each time-step’s hidden representation. The attention weights indicate how much attention or importance the model assigns to each time-step when making a prediction. (e.g., higher attention weights suggest that a particular time-step has a more significant influence on the current prediction, while lower weights show less relevance). The attention weights can be visualized to acquire insights into which time-steps the model considers most important for the forecast. By examining the weights, patterns, or trends in the input sequence that the model relies on to make predictions can be identified. A key advantage of attention mechanisms is their ability to adaptively adjust the attention weights for each prediction. Thus, the model can give more weight to recent or relevant time-steps while reducing the importance of less relevant ones. Interpreting growth stage importance can be achieved by considering temporal attention weights in the time domain. The model was implemented using individual sensing modality inputs (e.g., hyperspectral features and LiDAR features in isolation), in addition to early fusion multi-modality, allowing interpretation of the temporal attention weights on each modality in order to determine the dates to be used in the different scenarios.

#### 3.5.3 Multi-modal network for the different RS modalities

The multi-modal network consists of two modules described in Section 3.5.2, one for each of the RS modalities and a fusion module, as proposed in (Wang et al., 2020a). Gradient blending is used to blend the multiple loss functions in each module (Wang et al., 2020a) and avoid over-fitting by choosing the scaling of the weights, as the architecture of each module converges at different numbers of epochs. In the fusion module, as seen in **Figure 5C**, two dense layers are added using a sigmoid activation function to generate a single prediction value.

### 3.6 Genetic clustering, stratified sampling, and evaluation metrics

Six principal components derived from the original genetic marker data explained 35% of the variance from the high dimensionality genetic marker matrix. They were clustered via k-means unsupervised classification to develop balanced groupings for stratified sampling for the training, validation, and testing datasets. Performance was evaluated using R^2^ *_ref_* calculated as Equation (1) (relative to the one-to-one reference line) and the root mean squared error calculated as Equation (2) (RMSE):


(1) $$R_{ref}^{2}=\frac{\sum {\left(y_{i}-\hat{y_{i}}\right)}^{2}}{\sum {\left(y_{i}-\bar{y}\right)}^{2}}$$

(2) $$RMSE=\;\sqrt{\frac{\sum {\left(\hat{y_{i}}-y_{i}\right)}^{2}}{n}}$$

where $y_{i}$ is the observed yield reference value, $\hat{y_{i}}$ is the predicted yield value, and $\bar{y}$ is the mean observed value. The total number of samples is denoted by *n*.

## 4 Experimental results and discussion

### 4.1 Individual modality growth stages importance inferred from attention weights

**Figure 6:**
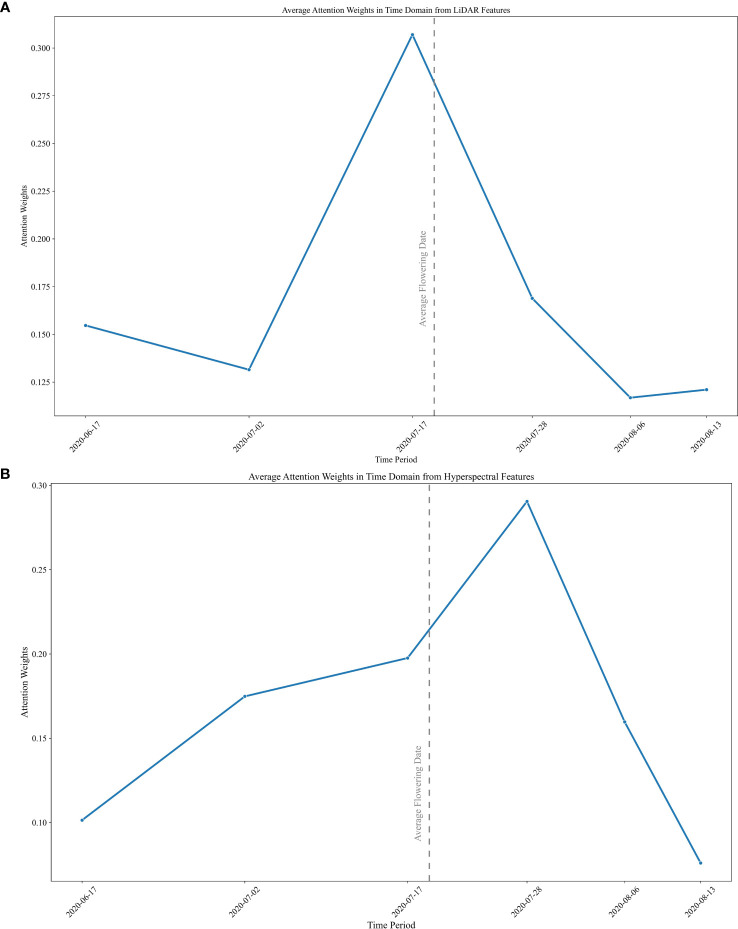
Average values of the attention weights in the time domain in **(A)** LiDAR and **(B)** hyperspectral modalities.

Plots of the attention weights within each time-step show the relative importance of the time periods for predicting end-of-year yields. The visualization also provides a useful connection between growth stages and RS data inputs. A heatmap plot of the attention weights was obtained by summing the feature weights within each time-step. Individual RS attention weights for each plot are shown in **Supplementary Figure 4**. Although the values of the attention weights vary across individual plot level predictions, a distinct trend is clear in the plot of the temporal weights averaged over all the plots. The relative importance of the LiDAR features during the early season is clear, while the impact of hyperspectral features on yield prediction was greater starting mid-season. The merged representation by the average weights of the testing dataset at each time-step is plotted in **Figure 6**. The average flowering date for the doubled haploid varieties is denoted by the dashed line. LiDAR attention weights show a peak value around the flowering time, while the maximum value of the attention weights for hyperspectral imagery is during the early grain filling stages, which coincides with the physiological growth characteristics of maize. In the early stages of growth, the plant prioritizes the utilization of nutrients for biomass growth. However, as it reaches the flowering stage, the plant undergoes a process of remobilization, redirecting its resources towards grain filling. This chemistry related transition can be observed in the hyperspectral imagery.

Based on the importance indicated by the attention weights, four scenarios were investigated for yield prediction models: (a) Using all six dates in both modalities; (b) using only 3 dates prior to mid-season in each modality; (c) using the first four dates with LiDAR data and the middle 4 dates for the hyperspectral data; (d) using three mid-season LiDAR dates and four mid-season hyperspectral dates. The goal was to investigate the contributions of the two sources of RS data throughout the growing season, while reducing the size of the network to include the most meaningful inputs.

### 4.2 Maize grain yield predictions

**Figure 7:**
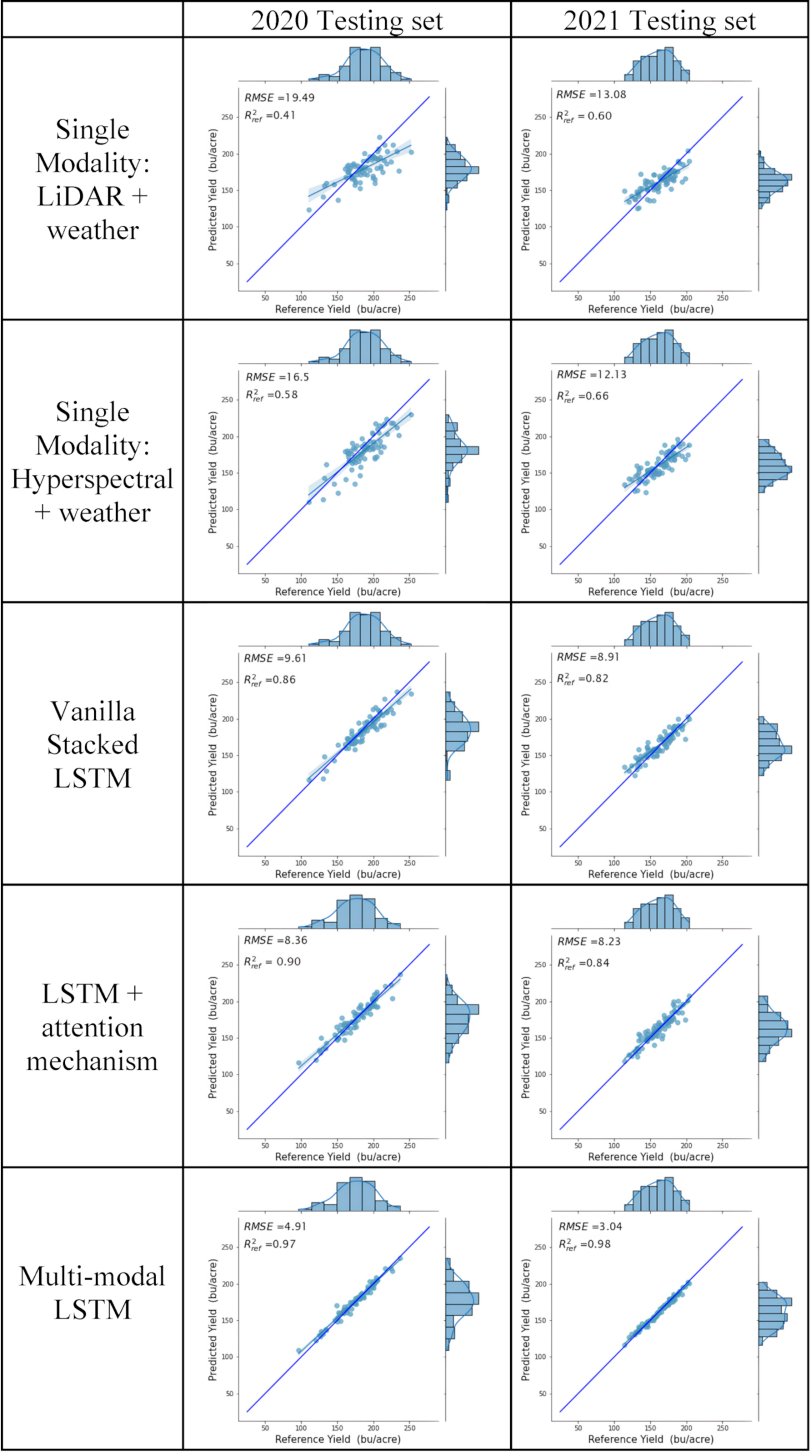
Model performance in deep learning networks developed for maize grain yield using the full season (**Table 2**) of RS data.

The yield forecast based on inputs from the individual modalities indicates that RS data can effectively function as a time series input to deep learning models during the growing season. Integrating these modalities, whether through early fusion in the initial deep learning models or through late fusion in the multi-modal network, leads to a significant enhancement in the prediction accuracy of the models. The same hyperparameters described in Section 2.5 were used to train and test all the scenarios in both years. **Figure 7** displays a comparison of the projected grain yields for Scenario 1 and the ground reference data. These results represent the best model from cross-validation; the results shown in **Table 4** include the sample mean and standard deviations from all the cross-validation predictions.

**Table 4:** Performance comparison of deep learning models for different scenarios.

	GxE 2020 Field 54				GxE 2021 Field 42			
	Independent Testing Data		Complete Dataset		Independent Testing Data		Complete Dataset	
	R^2^_ref_	RMSE	R^2^_ref_	RMSE	R^2^_ref_	RMSE	R^2^_ref_	RMSE
Scenario 1: All Dates								
**Stacked LSTM**	0.82 ± 0.13	9.50 ± 4.09	0.91 ± 0.06	6.57 ± 1.09	0.74 ± 0.22	11.88 ± 4.44	0.87± 0.04	8.71 ± 1.31
**Attention Network**	0.84 ± 0.10	8.36 ± 2.35	0.94 ± 0.05	8.06 ± 1.05	0.80 ± 0.14	11.46 ± 3.87	0.83 ± 0.09	8.42 ± 2.20
**Multi-modal Network**	0.89 ± 0.16	6.58 ± 3.04	0.96 ± 0.03	3.25 ± 1.25	0.87 ± 0.17	8.97 ± 4.02	0.96 ± 0.06	3.21 ± 1.99
Scenario 2: Predictions Based on Mid-season Data								
**Stacked LSTM**	0.64 ± 0.14	15.51 ± 2.49	0.80 ± 0.10	12.68 ± 1.32	0.63 ± 0.26	13.63 ± 2.78	0.83± 0.04	9.98 ± 1.31
**Attention Network**	0.79 ± 0.20	11.10 ± 5.94	0.89 ± 0.03	7.09 ± 1.91	0.74 ± 0.20	12.28 ± 5.44	0.78 ± 0.04	10.31 ± 1.32
**Multi-modal Network**	0.86 ± 0.06	8.02 ± 1.13	0.90 ± 0.04	6.60 ± 1.16	0.81 ± 0.15	10.02 ± 3.64	0.90 ± 0.04	6.94 ± 2.29
Scenario 3: Predictions based on Temporally Shifted LiDAR and Hyperspectral Datasets								
**Stacked LSTM**	0.77 ± 0.15	11.91 ± 4.64	0.88 ± 0.08	8.57 ± 1.59	0.71 ± 0.23	13.88 ± 6.22	0.87± 0.14	7.98 ± 1.31
**Attention Network**	0.82 ± 0.10	11.36 ± 2.35	0.92 ± 0.06	9.06 ± 2.05	0.78 ± 0.18	10.86 ± 3.87	0.83 ± 0.09	11.02 ± 1.20
**Multi-modal Network**	0.87 ± 0.16	7.68 ± 3.04	0.94 ± 0.02	5.75 ± 2.25	0.85 ± 0.12	8.01 ± 4.02	0.96 ± 0.06	3.21 ± 1.99
LiDAR {6/17/20, 7/2/20, 7/17/20, 7/28/20}; {7/3/21, 7/19/21, 7/27/21, 8/16/21} Hyperspectral: {7/2/20, 7/17/20, 7/28/20, 8/13/20}; {7/19/21, 7/27/21, 8/16/21, 9/6/21}								
Scenario 4: Predictions based on 3 Midseason LiDAR and 4 Midseason Hyperspectral Datasets								
**Stacked LSTM**	0.76 ± 0.21	12.02 ± 2.04	0.85 ± 0.09	7.64 ± 2.67	0.71 ± 0.05	13.24 ± 3.01	0.85± 0.09	9.05± 2.45
**Attention Network**	0.83 ± 0.16	10.94 ± 3.05	0.91 ± 0.10	9.65 ± 3.54	0.75 ± 0.02	12.01 ± 3.87	0.84 ± 0.12	10.74 ± 2.45
**Multi-modal Network**	0.85 ± 0.10	8.87 ± 2.45	0.95 ± 0.16	4.98 ± 3.01	0.84 ± 0.09	8.85 ± 2.74	0.94 ± 0.15	5.01 ± 3.45
LiDAR {6/17/20, 7/2/20, 7/17/20},; {7/3/21, 7/19/21, 7/27/21} Hyperspectral: {6/17/20, 7/2/20, 7/17/20, 7/28/20}; {7/3/21, 7/19/21, 7/27/21, 8/16/21}								

As shown in **Figure 7**, the original basic vanilla stacked LSTM model with full season data from both remote sensing modalities had significantly worse performance in both the RMSE and R^2^_ref_ values compared to other multi-modal LSTM models. The accuracy increased as the model architecture was enhanced by adding attention mechanisms. Based on the RMSE and R^2^_ref_ metrics, the multi-modal architecture, which was also integrated with attention mechanisms, successfully established temporal relationships, and captured inter-modal connections through independent processing of the RS sources.

Values of the evaluation metrics from all the scenarios are listed in **Table 4**. Comparing the results from 2020 to 2021, there was a slight decrease in model performance from 2020 to 2021 based on explained variance. One plausible reason for this could be the difference in GDD days between the RS dates for the two years. There was a minor misalignment between the dates of the RS datasets and the plants’ growth stages in 2021 due to a heavy precipitation period during tasseling. Remote sensing data acquisition was not possible on multiple days because of adverse weather conditions. This small reduction in model performance occurred across all the architectures. Alternatively, the difference can be attributed to the variation in grain yield performance observed in the same hybrids throughout both years, as depicted in **Figure 2B**. Despite identical hybrids being grown in both years, there is a noticeable disparity in the distributions of grain yield values. The significant discrepancy in precipitation between the two years, given that 2021 experienced above-average levels of precipitation, may be the underlying factor contributing to the difference in grain yield. To provide a more comprehensive representation of the metrics, **Supplementary Figure 5** includes a plot of the error bars derived from **Table 4**.

Results of the scenarios based on different dates for remote sensing data acquisitions shown in **Table 4** demonstrate that inclusion of all the dates in the networks yielded the most accurate predictions for all the model architectures. However, the prediction accuracies in most networks did not decrease significantly for the other scenarios that were based on subsets of time periods. The second scenario, which includes both modalities until mid-season, had a significant decrease in the value of R^2^_ref_. While the crop undergoes nutrient redistribution from biomass to grain filling by mid-season, the final grain yield is still influenced by key environmental characteristics and plant genetics in its reproductive stages. These results also illustrate the contribution of attention mechanisms in the networks, as they enable the model to learn data patterns and retain crucial features at each time-step. Among the scenarios based on subsets of the whole season data, Scenario 3 had the best performance. It used the time-steps associated with the average attention weights shown in the visualization. Compared to Scenario 2, the third scenario indicates that the combination of LiDAR and hyperspectral data acquired during periods when their individual explanatory capability is greatest, in combination with the multi-modal network architecture, can provide accurate predictions of maize grain yield. The results reinforce the complementary capability of the two technologies and are consistent with the crop physiology.

### 4.3 Integrative multi-modal RS for precision phenology: matching maize growth stages and dynamics

The results in Section 4.2 indicate that using multi-modal RS time series with either early fusion or late fusion techniques can effectively mimic the maize growing season by capturing sequential phenological features of the crop over time, corresponding to the different stages of growth. However, incorporation of late fusion resulted in enhanced accuracy and provided flexibility in remote sensing data collections. This could also improve model generalization, as all the remote sensing modalities are not required in each growth stage for future model implementations, but still yield adequate results.

Results of the study also support the following conclusions relative to LiDAR and hyperspectral RS data:

- Planting and emergence: LiDAR can capture the initial DTM that is important for estimating plant heights based on point clouds from later dates. Once the plants have emerged, hyperspectral imagery captures small green shoots, resulting in changes in the spectral signature of the field.
- Vegetative growth: As maize enters the vegetative stage, the increase in chlorophyll content and leaf area leads to a stronger absorption of energy in the red portion of the spectrum and greater reflection in the near-infrared, as shown in the hyperspectral indices. The increase in plant material during this time is also clearly indicated in the LiDAR metrics.
- Reproductive stage: The transition from vegetative to reproductive stages (tasseling, silking, and pollination) involves changes in both plant structure and chlorophyll. These changes can be detected through shifts in the spectral signatures captured in the time series data.
- Maturity: As maize reaches maturity, the plants undergo senescence, where the chlorophyll content in the leaves decreases and they transition from a green color to a more yellow-brown hue. During this stage, the plant nutrients begin to break down, and nitrogen, for example, is transferred from the leaves to support the filling of the grain. These changes are visible, particularly in hyperspectral imagery.

## 5 Final discussion and conclusions

This study investigated plot-level maize grain yield predictions through three LSTM-based RNN deep learning models, over two years of GxE experiments in Indiana. The models leveraged genotypic, remote sensing, and weather data in their predictions.

The advantages of integrating multi-modality remote sensing become evident when comparing the outcomes of single modality to those achieved by networks utilizing early fusion or late fusion multi-modality remote sensing data. The *R^2^_ref_* values ranged from 0.6 to 0.95, showcasing their ability to effectively model time series remote sensing and weather data. The multi-modal network provided the best results, especially when compared to the traditional vanilla stacked LSTM. Temporal attention allowed these models to focus on specific times during the growing season. By incorporating attention weights to assess the relevance of each time-step, a more comprehensive understanding of the model’s prediction mechanism was achieved. This insight can result in more accurate forecasting and provide valuable information on experiments of plots where *in situ* reference data can potentially be increased, and models can be enhanced. Despite the results from all scenarios providing predictions that are potentially useful for breeders in selecting specific varieties, Scenario 3 is notable with accuracies exceeding 0.8 *R^2^_ref_* using remote sensing only for dates that align closely with the physiological stages of maize. Furthermore, the last date for remote sensing was scheduled for early August, which could provide additional information for late season testing (e.g., prioritizing in-depth nutrient studies or a stay-green study on the most successful hybrid models).

In the context of multi-modal architectures, RS data acquired by different sensing modalities enables more comprehensive data analysis and interpretation. The contributions of sensors in capturing important characteristics of crop physiology vary throughout the season. The process of combining complementary modalities in either early fusion or late fusion allows mitigation of the weaknesses inherent in one by utilizing the strengths of another, ultimately resulting in more accurate and reliable. For instance, when there is cloud cover, optical data may encounter difficulties. However, LiDAR data are not affected by clouds, ensuring that data collection can proceed unhindered regardless of weather conditions. The model’s flexibility is a benefit, as it does not have to include data from each modality in every time-step. To conclude, utilization of multi-modal RS data provides a synergistic framework that enhances the capabilities of individual sensor types, ultimately leading to a more nuanced and thorough comprehension of observed processes, which is useful for both research and operational environments.

Through the G2F initiative, the GxE experiments offer a unique opportunity to develop predictive models by leveraging the genetic data and multiple environmental setups. The networks proposed for predicting maize grain yield are designed to provide end-of-season outcomes for individual years. Given the multiple geographic and environmental conditions encountered, current research is being conducted on the application of domain adaptation to forecast the yield of maize grain for a different year and potentially a different location using semi-supervised approaches.

## Data availability statement

The raw data supporting the conclusions of this article will be made available by the authors, on request.

## Author contributions

CA: Conceptualization, Formal analysis, Methodology, Writing – original draft, Writing – review & editing. MC: Conceptualization, Formal analysis, Methodology, Supervision, Writing – review & editing. MT: Supervision, Writing – review & editing.

## Supplementary material

Supplementary Figure 1 Scree plot of the explained variance of the individual principal components determine the appropriate number of principal components.

Supplementary Figure 2 Example of a hyperspectral orthomosaic, OSAVI image, and the non-vegetation pixel mask during flowering time.

Supplementary Figure 3 Example of the reconstructed LiDAR point cloud during flowering time.

Supplementary Figure 4 Heatmap plot of the attention weights was obtained by summing the feature weights within each time-step **(A)** LiDAR and **(B)** Hyperspectral.

Supplementary Figure 5 Plot of the error bars derived from **Table 4**.
