# Supplementary figures and images for: Integrating multi-modal remote sensing, deep learning, and attention mechanisms for yield prediction in plant breeding experiments
Source: Front Plant Sci. 2024 Jul 25;15:1408047. doi: 10.3389/fpls.2024.1408047 (PMC11306015; doi:10.3389/fpls.2024.1408047)

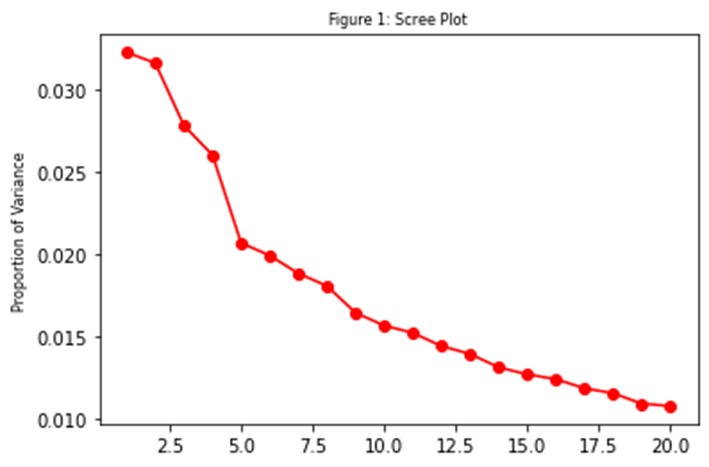

Supplement: Supplementary Figure 1 — Scree plot of the explained variance of the individual principal components determine the appropriate number of principal components. [file Image_1.jpeg]

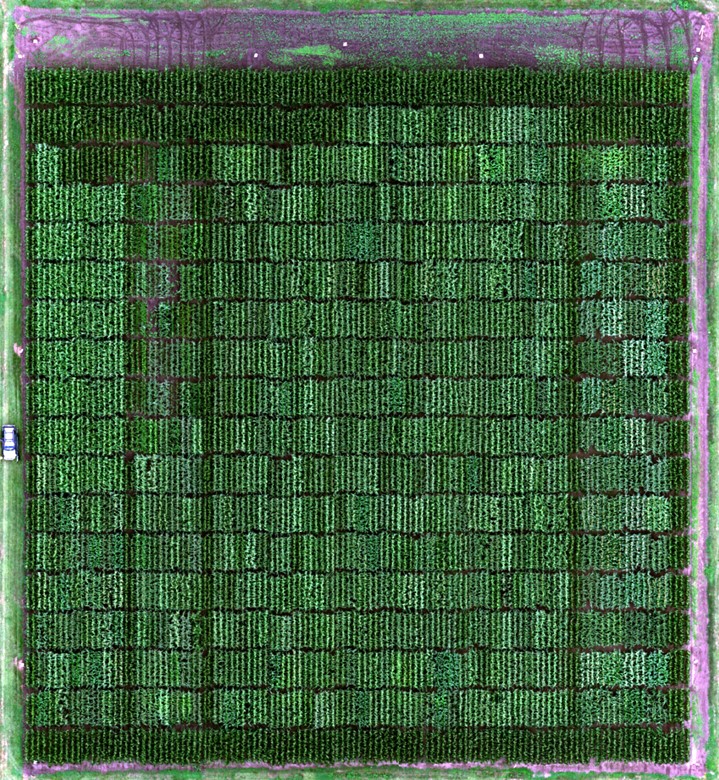

Supplement: Supplementary Figure 2 — Example of a hyperspectral orthomosaic, OSAVI image, and the non-vegetation pixel mask during flowering time. [file Image_2.jpeg]

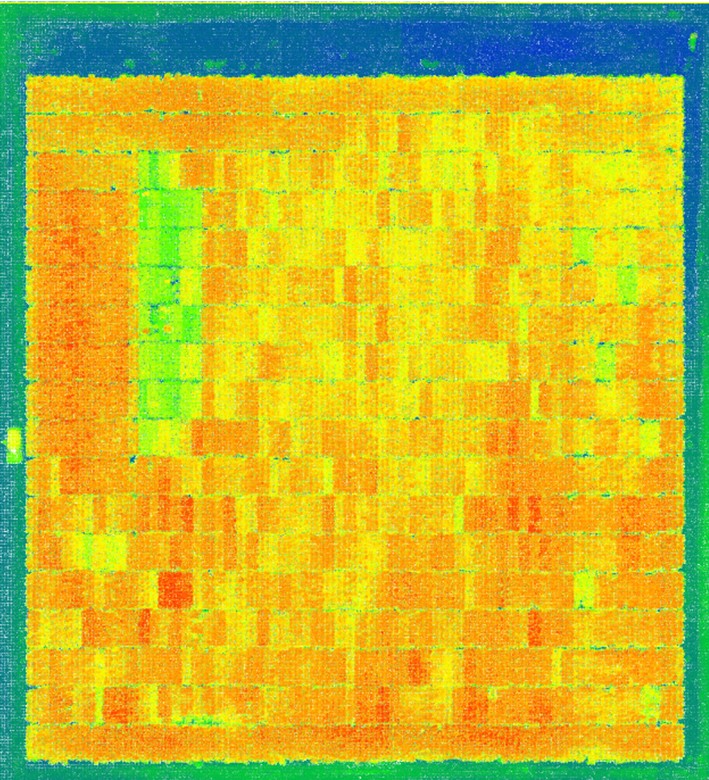

Supplement: Supplementary Figure 3 — Example of the reconstructed LiDAR point cloud during flowering time. [file Image_3.jpeg]

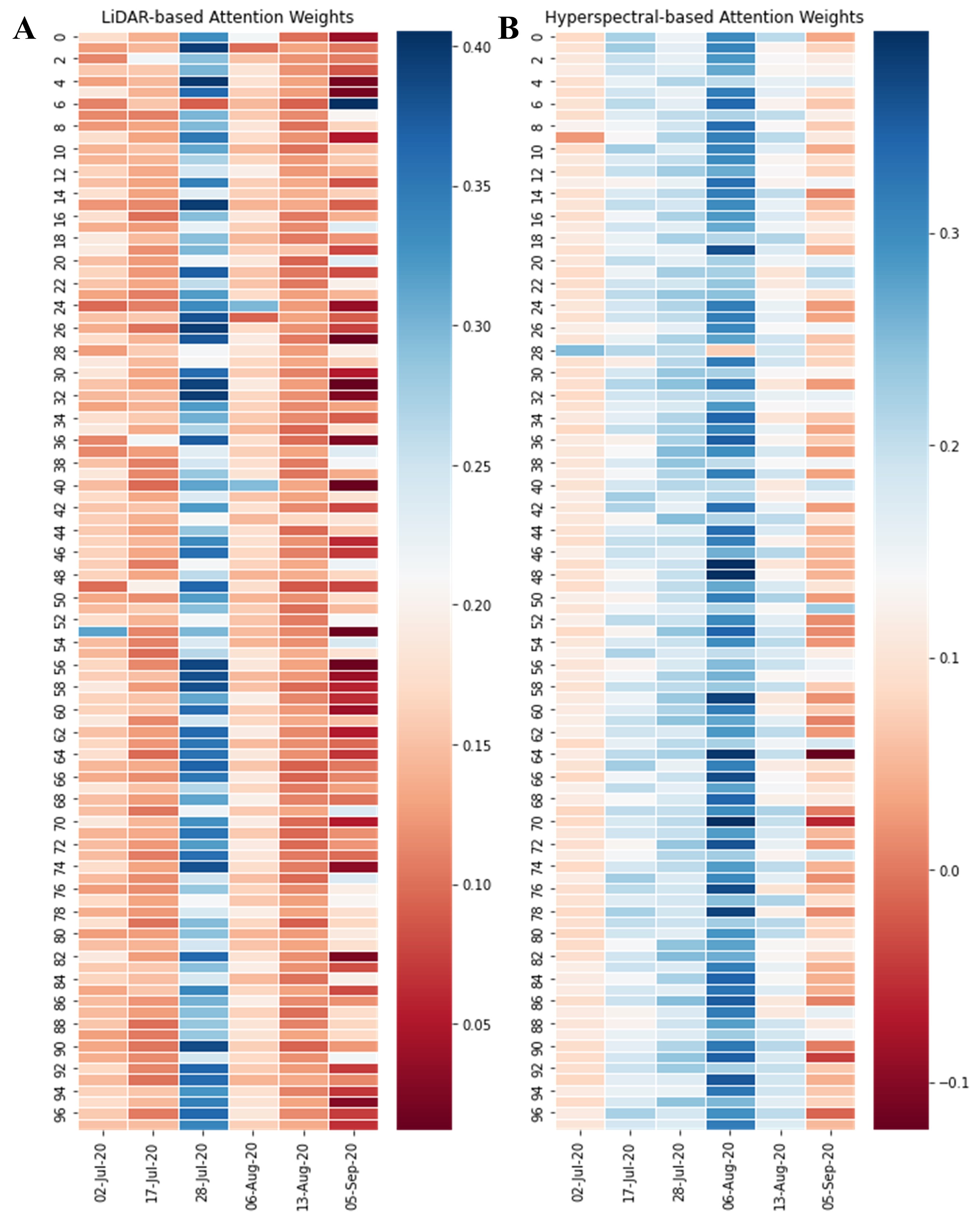

Supplement: Supplementary Figure 4 — Heatmap plot of the attention weights was obtained by summing the feature weights within each time-step (A) LiDAR and (B) Hyperspectral. [file Image_4.jpeg]

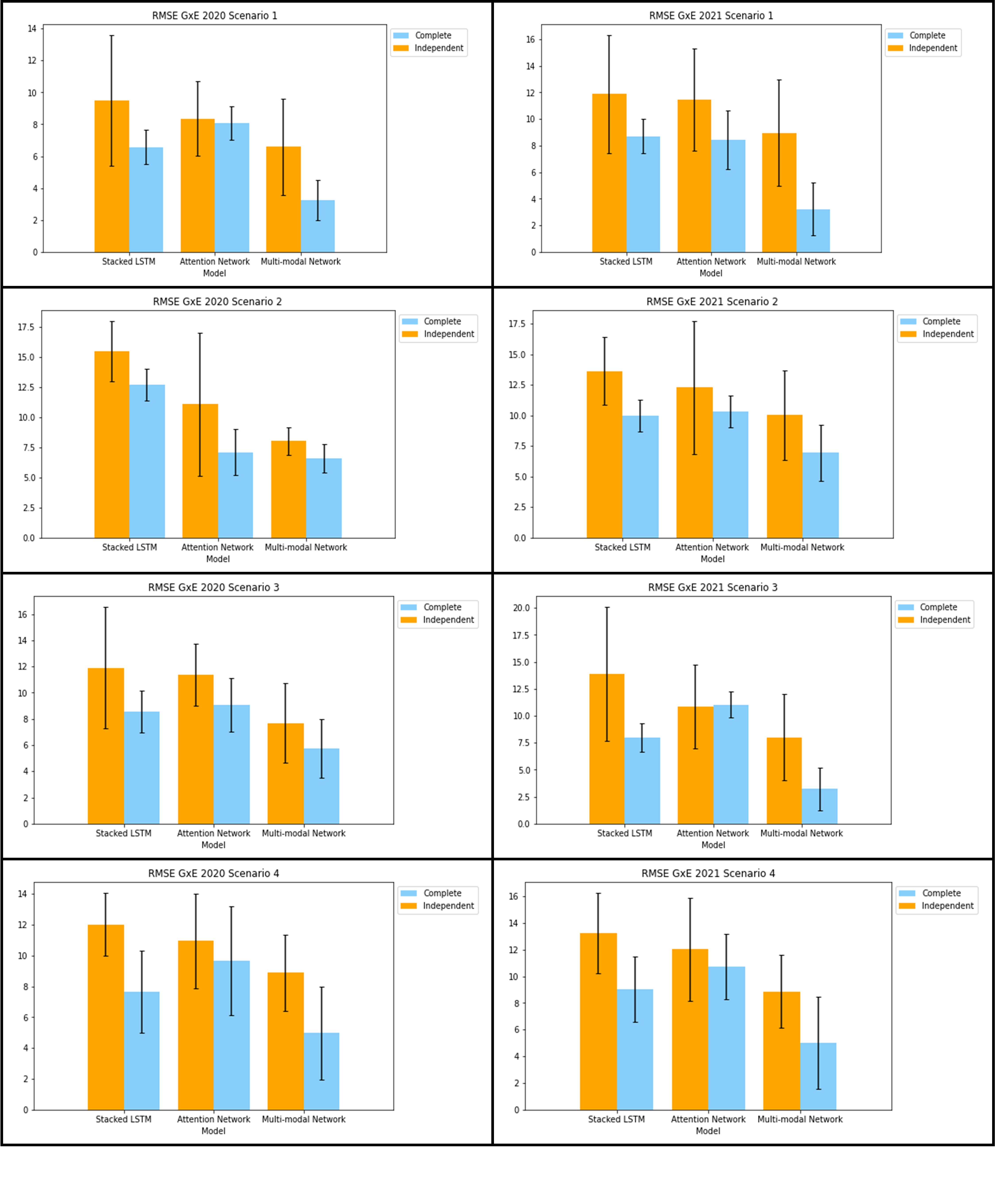

Supplement: Supplementary Figure 5 — Plot of the error bars derived from Table 4 . [file Image_5.jpeg]
